# A Scoring Tool to Identify East African HIV-1 Serodiscordant Partnerships with a High Likelihood of Pregnancy

**DOI:** 10.1371/journal.pone.0145515

**Published:** 2015-12-31

**Authors:** Renee Heffron, Craig R. Cohen, Kenneth Ngure, Elizabeth Bukusi, Edwin Were, James Kiarie, Nelly Mugo, Connie Celum, Jared M. Baeten, Partners PrEP Study, the Partners in Prevention HSV/HIV Transmission Study, and the Partners Demonstration Project Teams

**Affiliations:** 1 Department of Global Health, University of Washington, Seattle, United States of America; 2 Department of Epidemiology, University of Washington, Seattle, United States of America; 3 Department of Medicine, University of Washington, Seattle, United States of America; 4 Department of Obstetrics, Gynecology & Reproductive Sciences, University of California San Francisco, San Francisco, United States of America; 5 Jomo Kenyatta University of Agriculture and Technology, Nairobi, Kenya; 6 Microbiology Research, Kenya Medical Research Institute, Nairobi, Kenya; 7 Center for Clinical Research, Kenya Medical Research Institute, Nairobi, Kenya; 8 Department of Obstetrics & Gynecology, Kenya Medical Research Institute, Nairobi, Kenya; 9 Department of Reproductive Health, Moi University, Eldoret, Kenya; 10 Department of Obstetrics and Gynaecology, University of Nairobi, Nairobi, Kenya; University of Pittsburgh, UNITED STATES

## Abstract

**Introduction:**

HIV-1 prevention programs targeting HIV-1 serodiscordant couples need to identify couples that are likely to become pregnant to facilitate discussions about methods to minimize HIV-1 risk during pregnancy attempts (i.e. safer conception) or effective contraception when pregnancy is unintended. A clinical prediction tool could be used to identify HIV-1 serodiscordant couples with a high likelihood of pregnancy within one year.

**Methods:**

Using standardized clinical prediction methods, we developed and validated a tool to identify heterosexual East African HIV-1 serodiscordant couples with an increased likelihood of becoming pregnant in the next year. Datasets were from three prospectively followed cohorts, including nearly 7,000 couples from Kenya and Uganda participating in HIV-1 prevention trials and delivery projects.

**Results:**

The final score encompassed the age of the woman, woman’s number of children living, partnership duration, having had condomless sex in the past month, and non-use of an effective contraceptive. The area under the curve (AUC) for the probability of the score to correctly predict pregnancy was 0.74 (95% CI 0.72–0.76). Scores ≥7 predicted a pregnancy incidence of >17% per year and captured 78% of the pregnancies. Internal and external validation confirmed the predictive ability of the score.

**Discussion:**

A pregnancy likelihood score encompassing basic demographic, clinical and behavioral factors defined African HIV-1 serodiscordant couples with high one-year pregnancy incidence rates. This tool could be used to engage African HIV-1 serodiscordant couples in counseling discussions about fertility intentions in order to offer services for safer conception or contraception that align with their reproductive goals.

## Introduction

Pregnancy and the birth of healthy children are important aspirations for many couples, including those affected by HIV-1. For HIV-1 serodiscordant couples (i.e. couples in which one partner is HIV-1 infected and the other is not), the risk of HIV-1 transmission is heightened during pregnancy and the period leading up to pregnancy.[1–3] Despite the risk for HIV-1 transmission that accompanies pregnancy attempts—specifically, times when couples reduce or entirely forgo condom use—the achievement of fertility goals is paramount for couples; indeed, pregnancy rates among HIV-1 serodiscordant couples are similar to the general population.[4, 5] For HIV-1 serodiscordant couples with fertility goals, an early strategy is to engage them in discussion about their fertility desires and timing so that appropriate recommendations about safer conception services or effective contraception can be made. Key safer conception interventions for low resource settings include antiretroviral therapy (ART) use by the HIV-1 infected partner, pre-exposure prophylaxis (PrEP) for the HIV-1 uninfected partner and condomless sex limited to periods with peak fertility.[6, 7] Additional interventions, including fertility screening, diagnostic testing with treatment for genital infections, and vaginal self-insemination can further reduce risk and are consistent with a harm reduction approach.[8, 9]

Half of all new HIV-1 infections in sub-Saharan Africa are estimated to occur in stable heterosexual relationships, making HIV-1 serodiscordant couples a priority target population for HIV-1 prevention interventions [10, 11]. While strategies exist to minimize HIV-1 risk for HIV-1 serodiscordant couples planning pregnancy, a challenge lies in identifying couples who may soon become pregnant in order to initiate discussions about fertility desires. Timely counseling can help direct couples towards safer conception interventions (in the event that pregnancy is desired) or contraception (if pregnancy is not immediately desired). Worldwide, 40% of pregnancies are estimated to be unintended [12]. Public health systems may benefit from a simple tool that uses easy-to-capture information to identify couples likely to become pregnant. [13]

Clinical prediction tools have been developed to aid providers in identifying persons at risk for clinical outcomes, including, in reproductive health, pregnant women at risk of operative delivery and preeclampsia.[14, 15] However, a tool has not been developed to identify couples who are most likely to become pregnant. We used standardized clinical prediction methods to generate and validate a simple tool to identify heterosexual East African HIV-1 serodiscordant couples with increased likelihood of becoming pregnant in the next one year.[16, 17]

## Methods

Data from three prospectively followed cohorts of East African heterosexual HIV-1 serodiscordant couples were used to derive and externally validate a pregnancy prediction model. Couples included those with HIV-1 infected women at risk of HIV-1 transmission to male partners as well as those with uninfected women at risk of HIV-1 acquisition. Participants were ≥18 and sexually active and HIV-1 infected partners were not using ART at enrollment. Interviewer-administered standardized questionnaires were used to obtain information about demographics, medical history and symptoms, sexual behavior and contraceptive use. Across studies, participants received comprehensive HIV-1 prevention services including individual and couples counseling, free condoms and treatment of sexually transmitted infections (STI) at all visits.

In the *Partners PrEP Study (derivation cohort)*, pregnancy testing was conducted on a monthly basis for HIV-1 uninfected women and as clinically indicated during quarterly study visits for HIV-1 infected women. This cohort consisted of 4747 HIV-1 serodiscordant couples from 9 sites in Kenya and Uganda who were participating in a randomized clinical trial of daily oral pre-exposure prophylaxis (PrEP) for HIV-1 prevention [18, 19]. For HIV-1 uninfected women, routine visit procedures included HIV-1 testing, study drug dispensing, and adherence counseling; blinded study drug was withheld during pregnancy and breastfeeding. HIV-1 infected women underwent 6-monthly CD4 count and HIV-1 RNA testing.

In the *Partners in Prevention HSV/HIV Transmission Study (external validation cohort)*, pregnancy testing was conducted quarterly for the 3408 women from 14 sites in 7 East and southern African countries who were participating in this randomized trial of daily acyclovir to prevent HIV-1 transmission from partners dually-infected with HSV-2 and HIV-1 [20, 21]. HIV-1 infected partners completed monthly study visits for study drug dispensation and adherence counseling and HIV-1 uninfected partners completed quarterly study visits for HIV-1 testing. To validate the pregnancy prediction model with this cohort, data were restricted to the 1760 couples from Kenyan and Ugandan sites.

In the ongoing *Partners Demonstration Project (external validation cohort)*, pregnancy testing is conducted for all women at enrollment and as clinically indicated during quarterly follow-up visits. Information on fertility intention is collected for all participants through a standardized interviewer-administered questionnaire asking if the participant desires another child in the future and when he/she would like to have a future child. In this implementation science-driven delivery project of PrEP as a “bridge” to ART use, 1013 high risk HIV-1 serodiscordant couples from 4 sites in Kenya and Uganda are followed for up to 24 months to assess their use of PrEP in a time-limited fashion until the HIV-1 infected partner initiates and sustains ART use [22]. To validate the pregnancy prediction model with this cohort, we included data through December 2014.

### Score derivation

Using univariate Cox proportional hazards models, we identified enrollment demographic, medical, and sexual behavior characteristics from female and male partners of participants in the Partners PrEP Study that predicted the first occurrence of a new pregnancy using a p-value cutoff of 0.05. We limited consideration of possible predictors to baseline characteristics so that the final tool would use information that could be routinely captured during an initial clinic visit. Pregnancy was defined to begin on the self-reported date of last menstrual period (LMP), for most pregnancies, or for those without LMP data, by counting backwards from the date of delivery based using the reported gestational age that the pregnancy achieved. Follow-up time was censored after the occurrence of a woman’s first pregnancy and after one year in the study. Continuous variables were grouped into the most predictive categories using optimal cutpoints identified through signal detection receiver operating characteristic (ROC) curves.[23] Categories covering few integers were collapsed to ensure that the final scoring tool would be easy to apply in a clinic setting.

All factors identified as predictive in univariate analysis were combined into a multivariate Cox proportional hazards model and a fully stepwise sequence selection procedure was used to identify the most predictive combination of factors. When two co-linear factors were identified through univariate analysis, we chose the factor that would be most simple to obtain from a woman to ensure that the final model would be feasible to implement. The Akaike Information Criterion (AIC) was used to identify the most predictive model (with the lowest AIC). To derive the score value for each category within predictors, we divided each coefficient from the multivariate proportional hazards model by the lowest coefficient among all predictors and rounded to the nearest integer.

Once we identified the most predictive model and scores for each predictor category, we applied the score to each woman in the dataset and calculated her pregnancy likelihood score. Pregnancy incidence rates were calculated as the number of new pregnancies occurring within one year of follow-up divided by the total time accrued between enrollment and pregnancy or one year for women who did not become pregnant. Score categories were determined by collapsing adjacent score levels that had similar incidence rates. We used ROC analysis to calculate the area under the curve (AUC) with the score as the sole predictor of pregnancy.

### Validation

We used a 10-fold cross validation technique to check for internal consistency of AUC within the derivation cohort. For external validation, we applied the score to enrollment data from participants from Kenyan and Ugandan sites in the Partners in Prevention HSV/HIV Transmission Study and the Partners Demonstration Project and calculated the pregnancy score for each woman. For each validation dataset, we calculated the AUC with the score as the sole predictor of pregnancy and pregnancy incidence rates for each category of the score. Women pregnant at enrollment were excluded and follow-up time was censored at one year in both external validation datasets.

Assessing fertility intentions could be a simple alternative method to predict upcoming pregnancy. However, it is unclear if this would be as predictive as a compilation of factors incorporated into one tool due to the frequency of unintended pregnancies and often changing pregnancy goals. Using data from the Partners Demonstration Project, we examined the predictive ability of fertility intentions by calculating the AUC for pregnancy prediction with a binary variable of immediate fertility intention (desiring a child within 1 year) as the sole predictor.

All analyses were conducted using SAS 9.4 (Cary, NC) and public domain ROC5 (Department of Veteran’s Affairs and the National Institute of Aging of the United States of America). Protocols for each study were approved by the University of Washington Human Subjects Division and ethics review committees for each of the study sites. Participants provided written informed consent.

## Results

### Participant characteristics

In the Partners PrEP Study, 58.9% of couples had an HIV-1 infected woman, the median age of women was 31 (interquartile range [IQR]: 26–36), most couples had at least 1 child together, and the median duration of partnerships was 8 years (IQR: 4–15, Table 1). Women reported a median of 4 sex acts with their partner in the month prior to enrollment and approximately one-quarter reported at least one sex act with their partner that was unprotected by a condom. Of the 4,340 couples whose female partner was not pregnant at enrollment, 600 (13.8%) became pregnant during the first year of follow up and the pregnancy incidence was 15.0 (95% confidence interval [CI] 13.8–16.2) per 100 person-years.

**Table 1:** Characteristics of couples participating in studies used for score derivation and validation.

	Partners PrEP Study	Partners in Prevention HSV/HIV Transmission Study, Kenyan and Ugandan sites	Partners Demonstration Project
Number of couples	4340	1760	872
% with HIV-1 infected women	2555 (58.9%)	1191 (67.7%)	538 (61.7%)
Woman’s age, Median (IQR)	31 (26–36)	29 (25–35)	27 (23–33)
Women’s number of children, Median (IQR)	3 (2–5)	2 (1–4)	2 (1–3)
Couple’s number of children, Median (IQR)	2 (1–4)	1 (1–3)	0 (0–2)
Partnership duration, years, Median (IQR)	8 (4–15)	6 (3–11)	4 (1–8)
% Married or cohabiting	4292 (98.9%)	1717 (97.6%)	851 (97.6%)
Years of school completed by the woman, Median (IQR)	7 (3–8)	8 (6–10)	8 (6–11)
Sex acts between study partners, past month, Median (IQR)*	4 (2–8)	3 (2–6)	5 (3–10)
% couples having at least 1 sex act without a condom, past month*	1091 (25.2%)	475 (27.0%)	548 (62.8%)
% women reporting a non-study sexual partner, past month	37 (0.9%)	16 (1.0%)	12 (1.4%)
% women using effective contraception (injectable, oral, IUD, implant, or surgical method)	1776 (40.9%)	338 (19.2%)	273 (35.2%)
% women with an STI**	433 (10.3%)	157 (9.5%)	—
% of HIV-1 uninfected women with HSV-2 infection**	1398 (80.3%)	492 (88.8%)	—
% experiencing pregnancy during follow-up	600 (13.8%)	312 (17.7%)	141 (16.2%)
One year pregnancy incidence rate per 100 person-years (95% CI)	15.0 (13.8–16.2)	19.8 (17.6–22.0)	17.4 (14.0–20.7)

*Based on the woman’s report.

**Neisseria Gonorrhoea, Chlamydia trachomatis,Trichonomas vaginalis, or syphilis; Baseline STI and HSV-2 testing was not conducted within the Partners Demonstration Project.

### Score derivation model

**Fig 1:**
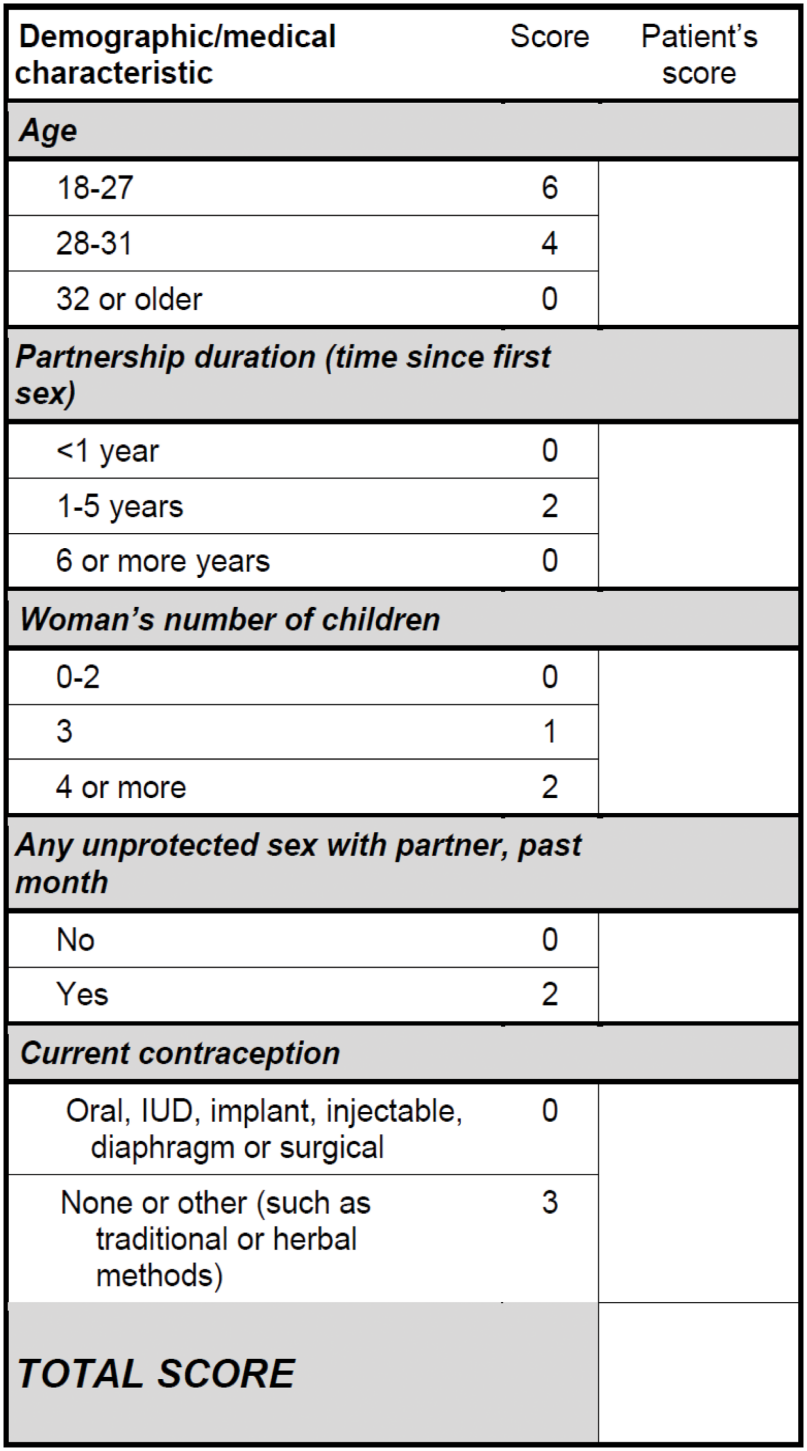
Pregnancy Likelihood Scorecard.

Univariate analysis identified multiple factors significantly associated with incident pregnancy within one year (Table 2). In a stepwise duration, woman’s number of children, having had sex unprotected by a condom in the past month, Cox proportional hazards multivariate model, five factors were retained for the final prediction model: woman’s age, partnership and non-use of an effective contraceptive. The highest value for an individual risk factor was 6 for women aged 18–27, with other factors scoring at lower values (Fig 1). Notably, HIV-1 status did not emerge as a key predictor.

**Table 2:** Predictors of pregnancy in the derivation cohort.

	Univariate models		Multivariate model		Stepwise multivariate model			
	HR (95% CI)	p-value	HR (95% CI)	p-value	HR (95% CI)	p-value	Regression coefficient	Score
***Age of woman (vs*. *≥32)***								
18–27	4.13 (3.36–5.09)	<0.001	4.87 (3.58–6.62)	<0.001	4.09 (3.22–5.19)	<0.001	1.41	6
28–31	2.38 (1.85–3.06)	<0.001	2.71 (1.94–3.78)	<0.001	2.50 (1.93–3.24)	<0.001	0.92	4
***Age of male partner (vs*. *≥28)***								
18–24	2.23 (1.72–2.90)	<0.001						
25–27	1.95 (1.52–2.50)	<0.001						
***Age difference (vs*. *male is 10 years older)***								
Male is younger	0.70 (0.52–0.95)	0.023	1.10 (0.72–1.67)	0.7				
Same age	0.55 (0.33–0.93)	0.024	0.77 (0.41–1.45)	0.4				
Male is 1–5 years older	0.86 (0.69–1.08)	0.186	1.07 (0.80–1.43)	0.7				
Male is 6–10 years older	0.98 (0.78–1.24)	0.885	1.19 (0.88–1.60)	0.3				
***Woman’s level of education*, *years (vs*. *≥5 years)***								
<1 year	0.88 (0.68–1.14)	0.341						
1–4 years	0.92 (0.74–1.14)	0.451						
***Partnership duration of 1–6 years (vs*. *<1 year or ≥6 years)***	2.31 (1.95–2.73)	<0.001	1.80 (1.41–2.30)	<0.001	1.58 (1.30–1.91)	<0.001	0.45	2
***Married or cohabiting (vs*. *no)***	1.36 (0.51–3.61)	0.537						
***Woman’s total number of children (vs*. *0–2 children)***								
3	0.79 (0.63–0.98)	0.035	1.42 (1.06–1.90)	0.02	1.29 (1.02–1.62)	0.0349	0.25	1
≥4	0.55 (0.45–0.66)	<.001	1.49 (1.11–2.00)	0.008	1.56 (1.24–1.96)	0.0002	0.44	2
***Number of children woman has with her male study partner (vs*. *0–1)***								
2	0.93 (0.75–1.16)	0.534						
≥3	0.53 (0.44–0.65)	<.001						
***Number of sex acts with male partner*, *past month***								
None	1.00							
1–4	1.28 (0.73–2.72)	0.39	1.54 (0.67–3.52)	0.3				
5–10	1.68 (0.95–2.99)	0.08	1.84 (0.80–4.25)	0.2				
11–18	1.71 (0.93–3.13)	0.84	1.90 (0.81–4.46)	0.1				
≥19	2.63 (1.38–5.02)	0.003	2.08 (0.81–5.29)	0.1				
***Condom use frequency with male partner*, *past month (vs*. *100% condom use)***								
No sex with study partner	0.76 (0.43–1.34)	0.343						
No condom use	1.63 (1.28–2.06)	<0.001						
Some condom use	1.50 (1.21–1.87)	<0.001						
***Unprotected sex with male partner*, *past month (vs*. *none)***	1.57 (1.32–1.87)	<0.001	1.45 (1.14–1.83)	0.003	1.63 (1.36–1.95)	<0.001	0.49	2
***Additional partner(s)*, *past month (vs*. *none)***	0.42 (0.11–1.61)	0.203						
***Male partner circumcised (vs*. *not circumcised)***								
Fully circumcised	0.93 (0.79–1.10)	0.398						
Partially circumcised	0.64 (0.15–2.62)	0.531						
***BV (vs*. *no BV)***	1.16 (0.95–1.42)	0.152						
***Woman has infection with gonorrhoea*, *chlamydia*, *trichomonas and/or syphilis***	1.19 (0.91–1.55)	0.20						
***Woman is HIV uninfected (vs*. *HIV-infected)***	0.61 (0.45–0.82)	<0.001	0.86 (0.64–1.15)	0.3				
***HSV-2 uninfected (HIV uninfected women only)***								
Indeterminate	1.49 (0.76–2.91)	0.248						
Positive	0.61 (0.44–0.85)	0.003						
***No use of effective contraception (vs*. *use of oral*, *injectable*, *IUD*, *implant or surgical)***	1.82 (1.52–2.19)	<0.001	1.74 (1.38 (2.20)	<0.001	1.92 (1.59–2.32)	<0.001	0.65	3

**Fig 2:**
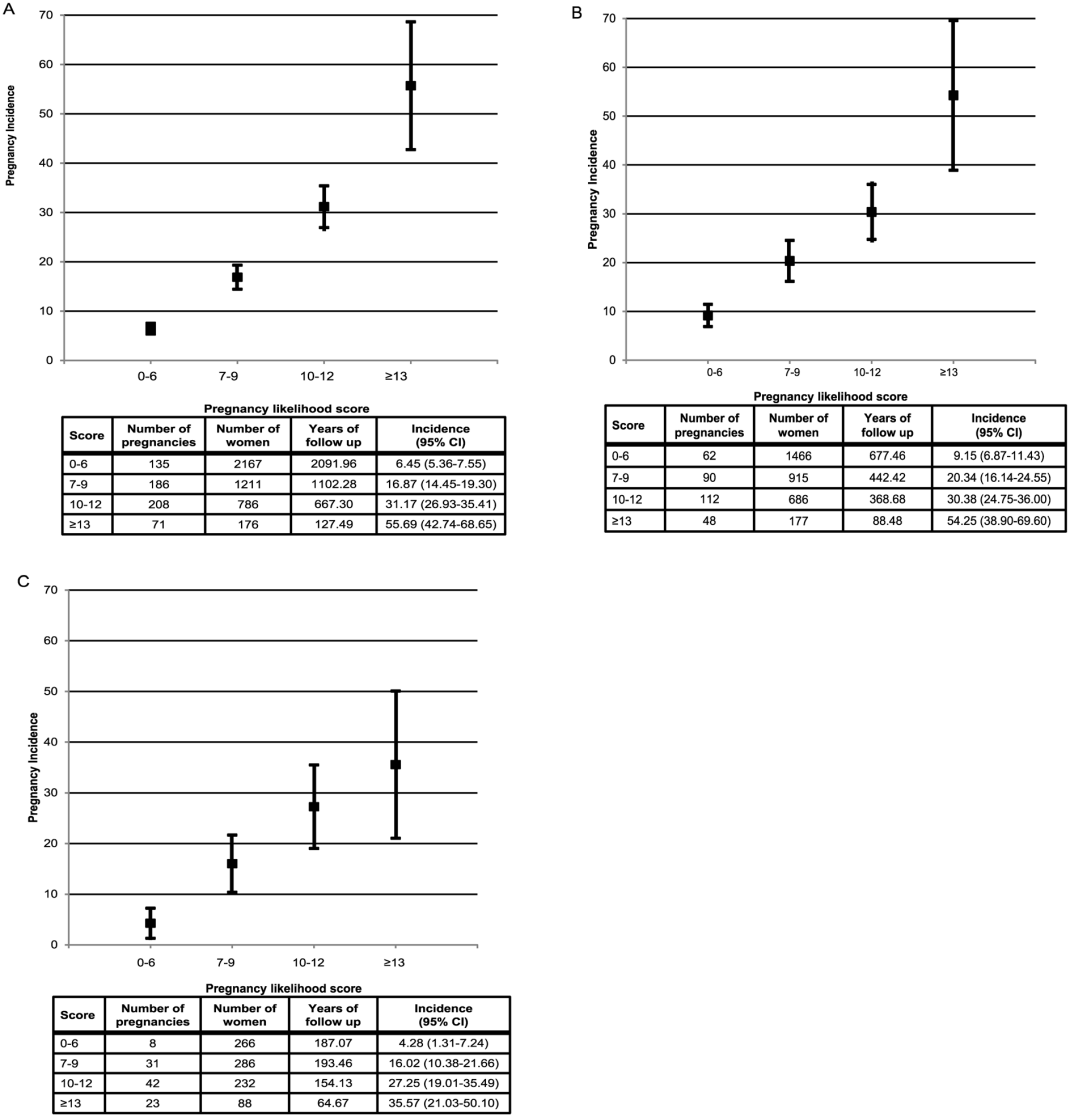
Pregnancy incidence rates by score among women in the A) Partners PrEP Study B) Partners in Prevention HSV/HIV Transmission Study at Kenyan and Ugandan sites and C) Partners Demonstration Project.

**Fig 3:**
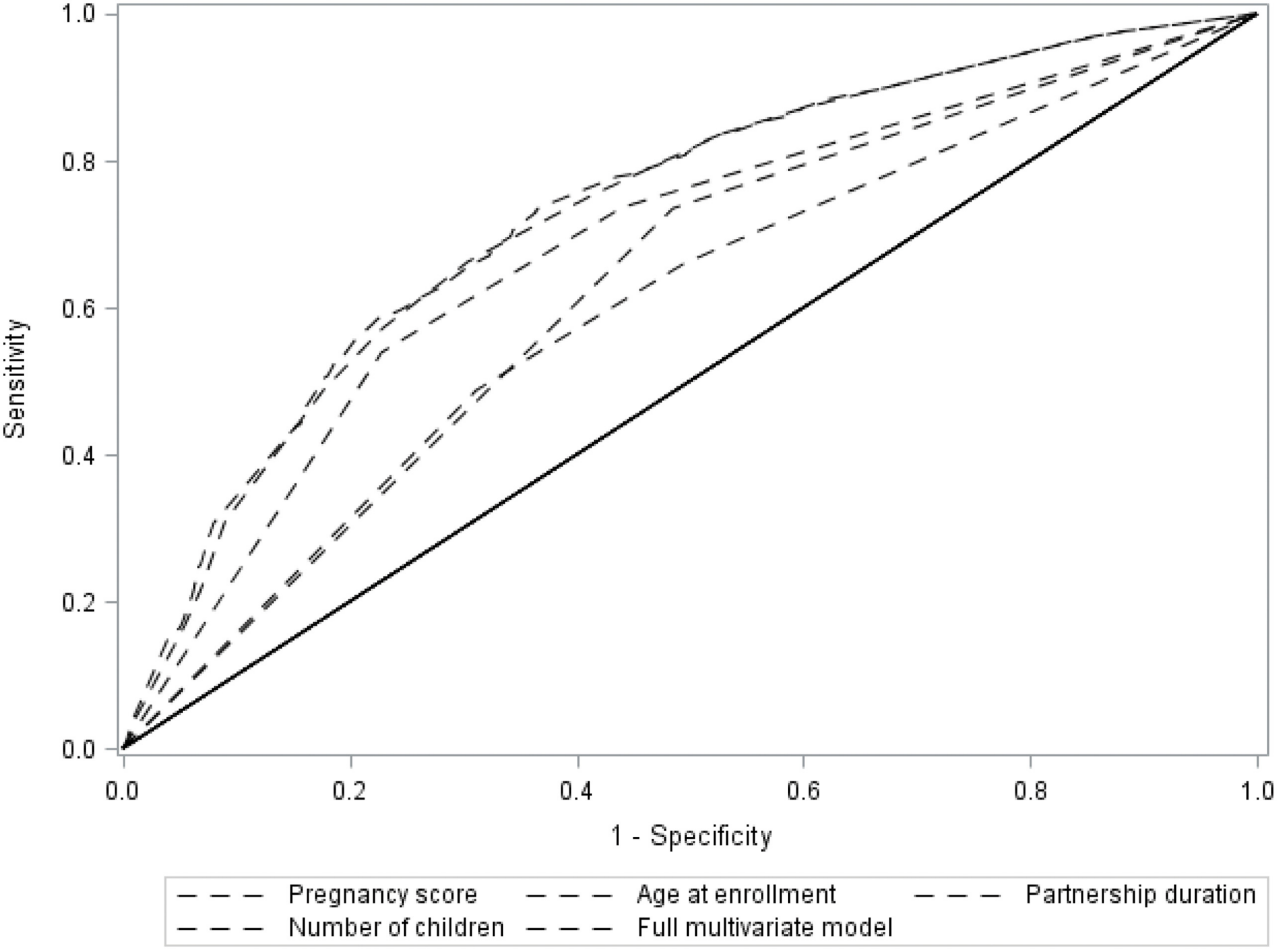
Receiver operating characteristic (ROC) curves for the pregnancy prediction score, individual continuous pregnancy predictors, and the multivariate model containing all predictors.

We applied the scores for each predictor to enrollment data from women in the Partners PrEP Study and calculated the pregnancy incidence for women in each level of the score. Half of the couples had a score of ≥7 and this score captured 78% of the pregnancies that occurred during follow-up; scores ≥13 predicted a pregnancy incidence >50% per year (Fig 2, panel A). The composite score had greater predictability than any of the individual factors alone (Fig 3). Among the categorical risk factors, having sex unprotected by a condom predicted only 33% of pregnancies. In ROC analysis, the score (as the sole predictor) and the stepwise multivariate model (with each predictor as a covariate) essentially overlapped, demonstrating that the score captured essentially all of the predictability of the multivariate model. The area under the curve (AUC) for the probability of the score to correctly predict pregnancy was 0.74 (95% CI 0.72–0.76). Ten-fold cross validation produced an average AUC of 0.75 (95% CI: 0.65–0.80), indicating the internal robustness of the prediction algorithm.

For external validation, we applied the score to Kenyan and Ugandan participants in the Partners in Prevention HSV/HIV Transmission Study and the Partners Demonstration Project. Characteristics of couples in the validation datasets were similar to the derivation cohort (Table 1) with trends towards younger ages, fewer children, and higher risk sexual behavior in the Partners Demonstration Project, which was designed to recruit couples at higher risk for HIV-1 transmission. [17, 24] The overall 12-month pregnancy incidence was 19.8 (95% CI 17.6–22.0) in the Partners in Prevention HSV/HIV Transmission Study and 17.4 (95% CI 14.0–20.7) in the Partners Demonstration Project.

In the Partners in Prevention HSV/HIV Transmission Study, 60.0% of the cohort had a pregnancy likelihood score ≥7 and couples in this range accounted for 250 (80.1%) of pregnancies that were experienced. In the Partners Demonstration Project, 69.5% of the cohort had a score ≥7 and couples in this range accounted for 92.3% of the pregnancies experienced. Thus, the score effectively identified a subset of each cohort with the highest pregnancy likelihood and the great majority of the pregnancies were experienced by this subset. When the score was applied to these external validation datasets, the AUC was 0.67 (95% CI 0.64–0.70) for the Partners in Prevention HSV/HIV Transmission Study and 0.67 (95% CI 0.63–0.71) for the Partners Demonstration Project.

The Partners Demonstration Project collected data at enrollment on fertility desires and intentions. Using immediate fertility intention as a sole predictor of pregnancy (indicated by saying “currently trying to get pregnant”) identified only 14% of pregnancies and the AUC was poor at 0.53 (95% CI 0.50–0.56); reporting immediate fertility intention or within the next 3 years accounted for 62.5% of the pregnancies, with an AUC of 0.60 (0.55–0.64).

## Discussion

We developed a pregnancy prediction scoring tool that can be used in research and clinical settings to identify HIV-1 serodiscordant couples that are likely to become pregnant within one year. In the current era of HIV-1 prevention, where antiretroviral interventions are incorporated into combination prevention strategies that nearly eliminate transmission within HIV-1 serodiscordant partnerships, efficient and cost-effective approaches are needed to identify and target couples who are at highest risk of transmission, including those likely to become pregnant.[6, 22] Couples often lack opportunities to discuss pregnancy desires with their care providers and many shy away from introducing the topic due to cultural stigma and the past guidance for HIV infected women to avoid pregnancy.[25] Providers could use this tool to engage HIV-1 serodiscordant couples in discussions about their fertility intentions, to empower women and their partners to determine when/if they want to have children and to receive the appropriate counseling and care that matches these desires, such as safer conception services or the provision of effective contraception. Notably, the pregnancy score appeared to be a better predictor than reported fertility intention. Thus, the tool could be used in conjunction with routine assessment of fertility desires to identify couples that might benefit most from clinician-initiated discussion about couple and individual fertility goals and counseling on how to achieve those goals. Depending on the clinic setting and goals, lower or higher cutpoints could be used to focus attention on couples with moderate, high, or extremely high pregnancy likelihood and balance the size of the population targeted with clinician time available for counseling.

Clinical prediction tools are useful to identify novel cohorts for research on a specific outcome or in a public health setting to triage individuals towards an individually-tailored intervention. In the ongoing Partners Demonstration Project, the use of a validated scoring tool for HIV-1 transmission successfully identified a high risk cohort with age, sexual behavior, and plasma viral load characteristics indicative of much greater HIV-1 risk than our previous cohorts.[24] Thus, in a research setting, these types of tools are useful and feasible to implement. Further operational research is needed to determine the feasibility of using these types of tools in a public health clinic setting where patient burden is greater and provider time is limited.

These data were from East African heterosexual HIV-1 serodiscordant couples and our results are most applicable to HIV-1 serodiscordant couples in that context, with similar pregnancy rates and fertility intentions. Importantly, our cohort did not include women who do not know their partner’s HIV-1 status, a group at potential risk for HIV-1 and pregnancy, and in urgent need of interventions to reduce HIV-1 risk that are integrated with pregnancy planning. Also, our cohort included only HIV-1 serodiscordant couples but couples that are HIV-1 seroconcordant (positive or negative) can benefit from open discussion and counseling with providers about fertility desires, pregnancy planning and their HIV-1 risks. We also do not know that all pregnancies were fathered by the male partner enrolled in the study, but reports of partnerships with men aside from those in the study were few and limiting follow-up to one year after study enrollment reduces this limitation. A strength of our methods was the use of multiple distinct cohorts to rigorously validate the prediction model and trends in pregnancy rates across categories of the score.

Pregnancy planning, pre-conception care, and open discussions with partners and providers are important, especially in the context of HIV-1 infection, to optimize pre-pregnancy health and birth outcomes. [26] The integration of discussions about fertility intentions into HIV-1 programs is urgently needed, especially for HIV-1 serodiscordant couples struggling to understand serodiscordance and to find ways to preserve their relationship in the midst of the risk of HIV-1 transmission. Our scoring tool could provide such an opportunity, to identify couples who have a high chance of becoming pregnant and increase dialogue about their best options for meeting their immediate and long term fertility goals with the lowest possible HIV-1 risk.
